# Elimination of Von Hippel-Lindau Function Perturbs Pancreas Endocrine Homeostasis in Mice

**DOI:** 10.1371/journal.pone.0072213

**Published:** 2013-08-20

**Authors:** Sapna Puri, Alejandro García-Núñez, Matthias Hebrok, David A. Cano

**Affiliations:** 1 Diabetes Center, Department of Medicine, University of California San Francisco, San Francisco, United States of America; 2 Servicio de Endocrinología y Nutrición, Instituto de Biomedicina de Sevilla, Hospitales Universitarios Virgen del Rocío/CSIC/Universidad de Sevilla, Sevilla, Spain; University of British Columbia, Canada

## Abstract

Mutations in the human homolog of the *Vhlh* gene [encoding the von-Hippel Lindau (VHL) protein] lead to tumor development. In mice, depletion of *Vhlh* in pancreatic ß-cells causes perturbed glucose homeostasis, but the role of this gene in other pancreatic cells is poorly understood. To investigate the function of VHL/HIF pathway in pancreatic cells, we inactivated *Vhlh* in the pancreatic epithelium as well as in the endocrine and exocrine lineages. Our results show that embryonic depletion of *Vhlh* within the pancreatic epithelium causes postnatal lethality due to severe hypoglycemia. The hypoglycemia is recapitulated in mice with endocrine-specific removal of *Vhlh*, while animals with loss of *Vhlh* predominantly in the exocrine compartment survive to adulthood with no overt defects in glucose metabolism. Mice with hypoglycemia display diminished insulin release in response to elevated glucose. Significantly, the glucagon response is impaired both *in vivo* (circulating glucagon levels) as well as in an *in vitro* secretion assay in isolated islets. Hypoxia also impairs glucagon secretion in a glucagon-expressing cell line in culture. Our results reveal a novel role for the hypoxia/HIF pathway in islet hormone secretion and maintenance of the fine balance that allows for the establishment of normoglycemia.

## Introduction

The role of hypoxia in ß-cell formation and function has recently gained considerable interest [1]. Central to the hypoxic response is the transcription factor hypoxia-inducible factor (HIF) whose activity is regulated by proteasomal degradation in the presence of oxygen, a process mediated by the von Hippel-Lindau (VHL) tumor suppressor-containing ubiquitin ligase complex [1]. Several studies, including our own, have uncovered a critical role for the HIF hypoxia response pathway in glucose homeostasis [2], [3], [4]. Deletion of *Vhlh* (the murine homolog of *VHL*) specifically in the pancreatic ß-cell lineage renders ß-cells unable to respond appropriately to elevated glucose, leading to severe glucose intolerance. Furthermore, hypoxia-responsive genes are upregulated in islets of pre-diabetic Zucker diabetic fatty (ZDF) and diabetic Goto-Kakizaki (GK) rats [5], [6]. An increase in HIF1α is also observed in islets of diabetic Goto-Kakizaki (GK) rats.

These studies clearly suggest a critical role for VHL/HIF signaling in ß-cell function, raising the question of whether aberrant HIF activation might interfere with the function of other pancreatic cell types. Total pancreas inactivation of *Vhlh* using a *Pdx-1-Cre* (pancreatic and duodenal homeobox gene-1 promoter) transgenic mouse line causes glucose intolerance, similar to what has been observed in mice with deletion of *Vhlh* in ß-cells, but no other pancreatic abnormalities are observed [3]. In contrast, *Vhlh* inactivation using a different *Pdx-1-Cre* strain causes neonatal lethality in mice. The cause of lethality in these mice is currently unknown since no obvious pancreatic abnormalities were observed [7]. To resolve these contradictory findings and to further investigate the function of VHL/HIF pathway in pancreatic cells, we inactivated *Vhlh* using three different pancreatic Cre lines. Our data show that mice with a *Vhlh*-deficiency in all pancreatic cells die perinatally due to severe hypoglycemia, which appears to result from a defect in glucagon secretion. In agreement with the mouse studies, glucagon-producing cells cultured under hypoxic conditions fail to secrete glucagon in response to reduced glucose levels. Altogether, these results suggest a more general role of the VHL/HIF pathway in endocrine function.

## Results

### *Vhlh* loss in endocrine precursors leads to perinatal lethality

**Figure 1:**
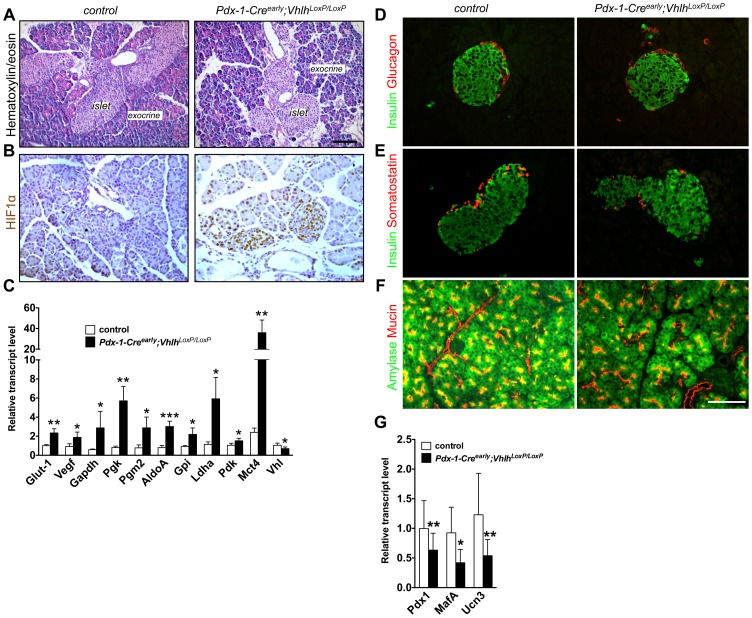
*Pdx-1-Cre^early^;Vhlh^LoxP/LoxP^* pancreata have alterations in metabolic and ß-cell genes but no change in morphology. **A**. Histological examination of pancreatic tissue from pups (p15) with Hematoxylin/Eosin staining shows the presence of normal exocrine and endocrine compartments in control and mutant mice. Size bar, 100 µm. **B.** Robust HIF1α accumulation is evident in pancreatic tissue (including endocrine, acinar and ductal cells) from *Pdx-1-Cre^early^;Vhlh^LoxP/LoxP^* mice (p15). **C.** Quantitative PCR was used to evaluate gene expression within the postnatal pancreas (p13-p18). HIF target genes including *Glut-1*, *Vegf*, glycolytic enzymes (*Gapdh*, *Pgk*, *Pgm2*, *AldoA*, and *Gpi*), and lactate biosynthetic genes (*Ldh*, *Pdk*, *Mct4*) were significantly upregulated, while *Vhlh* expression was reduced. Normalization of gene expression was to the housekeeping gene cyclophilin A. Five control and six mutant samples were analyzed. *p<0.05, **p<0.005, ***p<0.0005. Error bars represent standard deviation. Immunofluorescence confirms the expression of endocrine hormones insulin (**D,** **E**, green), glucagon (**D**, red), and somatostatin (**E,** red), and exocrine markers amylase (**F,** green) and mucin (**F,** red) in control and mutant tissues. Size bar, 50 µm. **G.** Gene expression of canonical ß-cell genes was evaluated using quantitative PCR in islets isolated from the postnatal pancreas (p13-p18). Markers of ß-cell maturity including *Pdx-1*, *MafA* and *Urocortin3* were reduced in their expression levels. Normalization of gene expression was to the housekeeping gene cyclophilin A. Six control and six mutant samples were analyzed. *p<0.05, **p<0.005, ***p<0.0005. Error bars represent standard deviation.

*Pdx-1-Cre^early^*, a line with homogeneous Cre expression in all pancreatic cell types in our hands, was crossed into the *Vhlh^loxP/loxP^* mouse [8], [9]. In agreement with a previous report, approximately 70% of *Pdx-1-Cre^early^;Vhlh^LoxP/LoxP^* pups died around weaning age (Figure S1A) [7]. Gross morphological analyses of pancreata from two week old *Pdx-1-Cre^early^;Vhlh^LoxP/LoxP^* pups did not reveal any apparent abnormalities (Figure 1A). Efficient excision of the *Vhlh^loxP/loxP^* allele by Cre recombinase was evidenced by significant accumulation of HIF1α in islet, acinar and ductal compartments of the pancreata from *Pdx-1-Cre^early^;Vhlh^LoxP/LoxP^* animals (Figure 1B). Increased expression of HIFα targets (a functional readout for HIF stabilization) was observed in pancreata from *Pdx-1-Cre^early^;Vhlh^LoxP/LoxP^* pups (Figure 1C). *Vhlh* expression was also reduced in these samples, correlating well with a robust activation of the hypoxic response pathway (Figure 1C). Expression of endocrine (insulin, glucagon and somatostatin) and exocrine (amylase and mucin) markers appeared normal by immunostaining (Figure 1D-F). Importantly, gene expression of ß-cell maturity markers *Pdx-1*, *MafA* and *Urocortin3* was reduced in mutant islets isolated from animals aged between p13 and p18 as compared to control littermates (Figure 1G). Thus, although there appeared to be delayed maturation of ß-cells, lethality was not due to an overt perturbation in pancreas formation.

**Figure 2:**
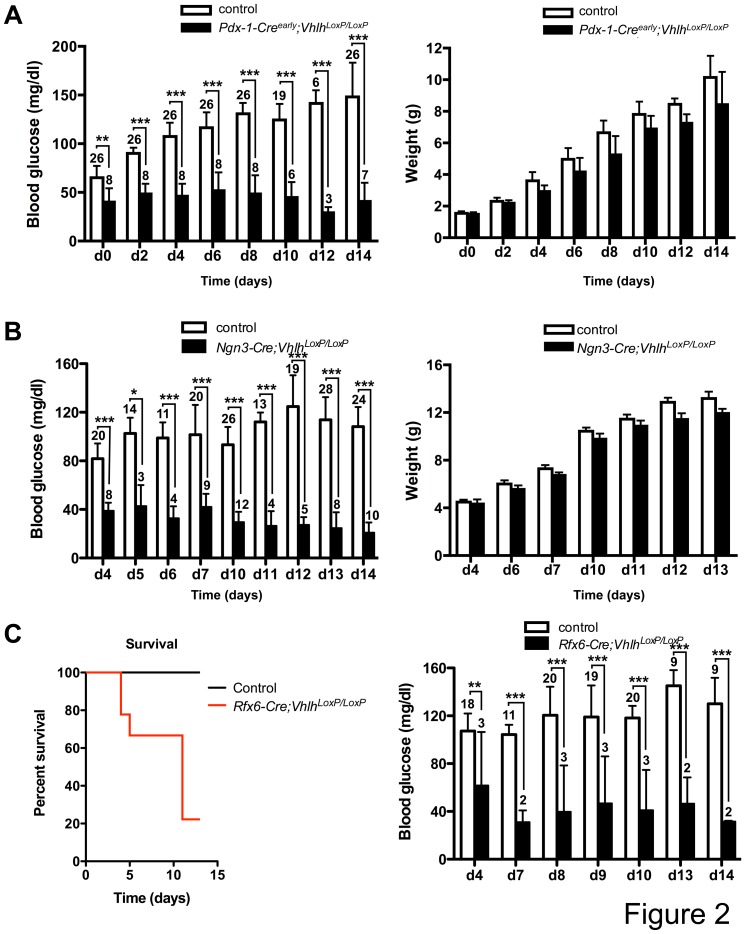
Defective insulin secretion in islets depleted of *Vhlh*. **A** ****. Left, VHL depletion in pancreatic progenitors (*Pdx-1-Cre^early^;Vhlh^LoxP/LoxP^*) leads to severe hypoglycemia (black bars) while control littermates (open bars) are normoglycemic. N numbers are noted on the bar graphs. Right, comparison of weight gain between control (open bars) (n = 5) and *Pdx-1-Cre^early^;Vhlh^LoxP/LoxP^* (black bars) (n = 6) pups. **B**. Left, VHL depletion in endocrine progenitors (*Ngn3-Cre;Vhlh^LoxP/LoxP^*) leads to severe hypoglycemia (black bars) while control littermates (open bars) are normoglycemic. N numbers are noted on the bar graphs. Right, comparison of weight gain between control (open bars) and *Ngn3-Cre;Vhlh^LoxP/LoxP^* (black bars) pups. Four control and three mutant animals were analyzed for the *Ngn3-Cre;Vhlh^LoxP/LoxP^* cohort. **C**. Left, Survival curve of *Rfx6-Cre;Vhlh^LoxP/LoxP^* mice (n = 9) compared to control (n = 27) littermates. Right, VHL depletion in pancreatic progenitors (*Rfx6-Cre;Vhlh^LoxP/LoxP^*) leads to severe hypoglycemia (black bars) while control littermates (open bars) are normoglycemic. N numbers are noted on the bar graphs. *p<0.05, **p<0.005, ***p<0.0005. Error bars represent standard deviation.

Mice with *Vhlh* loss in ß-cells exhibit glucose intolerance [2], [3], [4], and the reduced expression of maturity markers led us to hypothesize that the perinatal lethality observed in *Pdx-1-Cre^early^;Vhlh^LoxP/LoxP^* mice might result from perturbed glucose homeostasis due to compromised cellular function. Surprisingly, blood glucose measurements in the postnatal period revealed that *Pdx-1-Cre^early^;Vhlh^LoxP/LoxP^* mice are severely hypoglycemic (Figure 2A). Two lines of evidence suggest that hypoglycemia is the cause and not the consequence of VHL-associated lethality. First, hypoglycemia is observed shortly after birth before the mutants display reduced weight gain compared to control littermates (Figure 2A). Second, the severity of hypoglycemia positively correlates with the poorest survival rates, as pups with milder hypoglycemia live longer (data not shown). To identify pancreatic cell type(s) responsible for the observed perinatal lethality and hypoglycemia in *Pdx-1-Cre^early^;Vhlh^LoxP/LoxP^* mice, *Vhlh* was specifically inactivated in the endocrine lineage using the *Ngn3-Cre* mouse strain, as *Ngn3* marks all endocrine progenitors during embryogenesis [8]. Significantly, *Ngn3-Cre;Vhlh^LoxP/LoxP^* mice displayed postnatal lethality similar to that observed in *Pdx-1-Cre^early^;Vhlh^LoxP/LoxP^* mice, with >75% pups exhibiting blood glucose levels below 20 mg/dl between two and three weeks of age (Figure 2B and data not shown). Endocrine hormones were expressed appropriately in the pancreata of *Ngn3-Cre;Vhlh^LoxP/LoxP^* mice (data not shown). Recently, concerns have been raised over the extra-pancreatic expression, especially in areas of the brain, of both *Pdx-1-Cre^early^* and *Ngn3-Cre* transgenic lines [10], [11]. A third Cre-expressing mouse line was employed to tease out the effect of loss of *Vhlh* in the pancreas. *Rfx6* is a transcription factor that is expressed in the gut endoderm during early development and is later restricted to islets in the pancreas, with no confounding brain expression [12]. *Rfx6-Cre;Vhlh^LoxP/LoxP^* pups exhibited severe postnatal hypoglycemia with associated lethality (Figure 2C). In fact, 50% of the *Rfx6-Cre;Vhlh* animals are dead by day 10 post-birth while the same is true closer to day 18 after birth for the *Pdx-1-Cre^early^;Vhlh* mice (Figure 2C and Figure S1A). Islet architecture was unaffected in the postnatal pancreas of *Rfx6-Cre;Vhlh^LoxP/LoxP^* pups (Figure S1B). Summarily, our results indicate that VHL loss in endocrine cells during embryogenesis leads to neonatal hypoglycemia and perinatal lethality.

It has been proposed that reduced survival in *Pdx-1-Cre^early^;Vhlh^LoxP/LoxP^* mice might be associated with exocrine defects [7]. Cre expression in the *Ptf1a-Cre* line [13] is low in endocrine cells, but robust in acinar cells and a subset of ducts (Figure S2A) [14], thus providing a useful tool to inactivate *Vhlh* predominantly in the exocrine compartment. Immunohistochemistry for HIF1α confirmed efficient inactivation of *Vhlh* in the exocrine, but not in the endocrine, compartment (Figure S2B). *Ptf1a-Cre;Vhlh^LoxP/LoxP^* mice survived to adulthood (>6 months) without any symptoms of compromised health (data not shown), demonstrating that exocrine ablation of VHL does not cause lethality. Notably, *Ptf1a-Cre;Vhlh^LoxP/LoxP^* animals were normoglycemic at all stages analyzed (Figure S2C). Thus, *Vhlh* inactivation in endocrine cells, not in the exocrine compartment, causes perinatal lethality.

### VHL permits appropriate hormone secretion within the pancreatic islet

**Figure 3:**
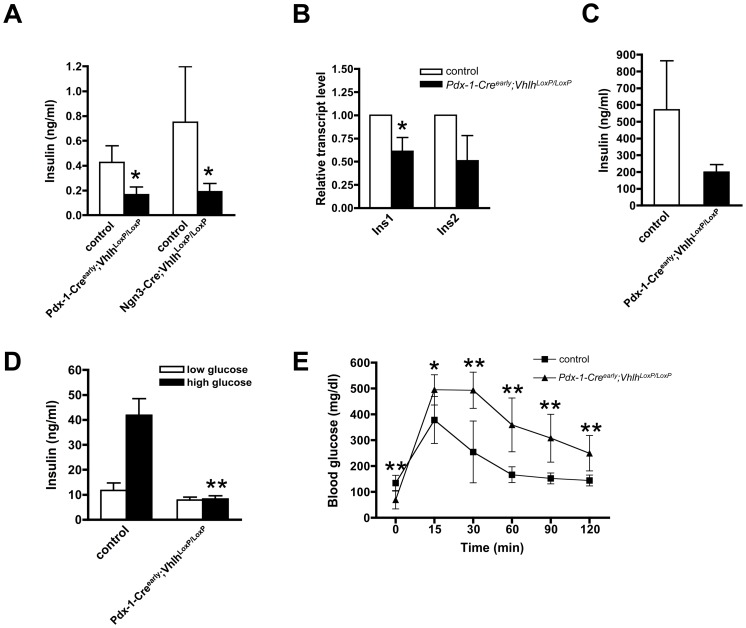
VHL inactivation impairs insulin secretion but does not result in hyperinsulinemia. **A**. Plasma insulin under fed conditions is reduced in *Pdx-1-Cre^early^;Vhlh^LoxP/LoxP^* (n = 3) and *Ngn3-Cre;Vhlh^LoxP/LoxP^* (n = 5) animals as compared to control animals (n of 4 and 5, respectively). *p<0.05. **B**. Transcript analysis in islets isolated from p15 pups (n = 3) by quantitative PCR reveals a moderate yet significant reduction in the *Ins1* transcript and a similar trend in *Ins2* transcript level in *Pdx-1-Cre^early^;Vhlh^LoxP/LoxP^* animals (black bars). **C**. Total insulin content appears reduced in islets isolated from *Pdx-1-Cre^early^;Vhlh^LoxP/LoxP^* (black bars, n = 4) versus control (open bars, n = 4) animals. **D**. In an *in vitro* secretion assay, islets isolated from control animals (n = 4) respond appropriately to a high glucose challenge, while *Pdx-1-Cre^early^;Vhlh^LoxP/LoxP^* islets (n = 4) are severely impaired in glucose stimulated insulin secretion. **p<0.005. **E**. An IPGTT on animals (p19–20) reveals that the *Pdx-1-Cre^early^;Vhlh^LoxP/LoxP^* animals (n = 8) are impaired in clearing glucose from the blood, while the control cohort (n = 16) normalizes blood glucose by the end of two hours. *p<0.05, **p<0.005. Error bars represent standard deviation.

One obvious cause of hypoglycemia is hyperinsulinemia. Surprisingly, plasma insulin was lower in *Pdx-1-Cre^early^;Vhlh^LoxP/LoxP^* and *Ngn3-Cre;Vhlh^LoxP/LoxP^* pups than in control littermates (Figure 3A). Given the overlapping phenotypes of the three mouse models, we focused subsequent analyses on the *Pdx-1-Cre^early^;Vhlh^LoxP/LoxP^* mice. Insulin content within the islet, measured by gene expression and total protein appeared reduced in the *Vhlh* deficient mice (Figure 3B-C). Insulin secretion from isolated islets showed that, while basal secretion was unaffected, there was a failure of hormone secretion upon exposure to increased glucose in *Vhlh* mutant mice (Figure 3D). In support of these data, mutant pups showed delayed glucose clearance in response to an intra-peritoneal glucose tolerance test, despite being hypoglycemic at the start of the assay (Figure 3E). Thus, overt hyperinsulinemia does not appear to cause hypoglycemia in *Pdx-1-Cre^early^;Vhlh^LoxP/LoxP^* pups.

**Figure 4:**
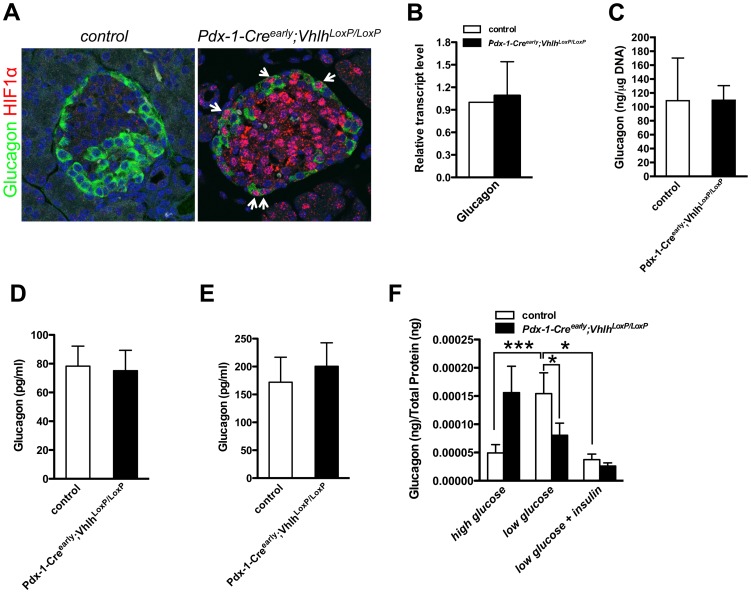
VHL inactivation impairs glucagon secretion. **A**. Robust HIF1α accumulation is evident in glucagon-expressing cells (arrowheads) of *Pdx-1-Cre^early^;Vhlh^LoxP/LoxP^* mice. **B.** Glucagon expression, as quantified by quantitative PCR in islets isolated from control or *Pdx-1-Cre;Vhlh^LoxP/LoxP^* animals (n = 3), was unchanged. Error bars represent standard deviation. **C**. Total glucagon content of islets is comparable between the two groups (n = 4). Error bars represent standard deviation. **D**. Circulating plasma glucagon in fed conditions is similar between control (open bars, n = 6) and *Pdx-1-Cre^early^;Vhlh^LoxP/LoxP^* (black bars, n = 6) mice. Error bars represent standard error of the mean. **E**. Circulating plasma glucagon after an overnight fast is similar between control (open bars, n = 8) and *Pdx-1-Cre^early^;Vhlh^LoxP/LoxP^* (black bars, n = 7) mice. Error bars represent standard error of the mean. **F**. Incubation of isolated islets in high followed by low glucose concentration led to glucagon secretion from control islets (n = 20-21), while *Pdx-1-Cre^early^;Vhlh^LoxP/LoxP^* islets appeared blocked in their ability to secrete glucagon (n = 21). *p<0.05, ***p<0.0005. Exogenous insulin (17 nM) led to a reduction in the glucagon secretion response from the control islets (n = 8) in the presence of low glucose but had no significant effect on the mutant islets (n = 8). Error bars represent standard error of the mean.

An alternative explanation for hypoglycemia could be compromised glucagon function. Mutant mice with defective α-cell formation or reduced glucagon production are severely hypoglycemic and display neonatal lethality [15], [16], [17]. Robust nuclear HIF1α accumulation was observed in α-cells of *Pdx-1-Cre^early^;Vhlh^LoxP/LoxP^* islets (Figure 4A). Quantification of HIF1α-Glucagon costaining revealed an ∼41% overlap. However, this number is likely an underestimation of α-cells with active HIF pathway, as cells with cytoplasmic HIF1α accumulation were not scored. Pancreatic control samples were clearly negative for HIF1α staining. No overt defects in α-cell formation, glucagon expression or glucagon production were observed in *Pdx-1-Cre^early^;Vhlh^LoxP/LoxP^* mice (Figure 1D and 4B-C). Surprisingly, serum glucagon levels in mutant mice under both fed conditions as well as after an overnight fast were similar to control littermates, pointing to a defective glucagon response, as persistent hypoglycemia should elicit an increase in circulating glucagon (Figure 4D-E). Administration of exogenous glucagon induced a comparable rise in blood glucose in mutant and control mice, indicating that glucagon sensitivity was normal in *Pdx-1-Cre^early^;Vhlh^LoxP/LoxP^* mice (Figure S3) and that hypoglycemia was not due to impaired liver response to glucagon, but most likely a result of deficient glucagon secretion. In support of this hypothesis, islets isolated from *Pdx-1-Cre^early^;Vhlh^LoxP/LoxP^* pups did not respond appropriately upon exposure to reduced glucose concentration *in vitro* (Figure 4F). Under conditions of high glucose, mutant islets appeared to secrete higher levels of glucagon when compared to the control islets. However, this difference was not statistically significant (p value = 0.067). On the contrary, under conditions of low glucose exposure, the mutant islets secreted less glucagon as compared to the control islets (p value = 0.04). Insulin plays an inhibitory role in glucagon secretion, and we tested the role that the reduction in insulin secretion from *Vhlh* mutant islets might play in glucagon secretion. Insulin was added to control and mutant islets upon incubation under low glucose conditions (Figure 4F). We find that upon incubation of control islets under low glucose with insulin, a suppression of glucagon secretion is detectable (p value = 0.02). In the mutant islets, however, there is no significant change in the levels of glucagon secretion with or without insulin under low glucose (p value = 0.141). These results indicate that insulin does not inhibit glucagon secretion in *Vhlh*-deficient islets, and argue that the defect in glucagon secretion in the *Vhlh* deficient mice is not an indirect effect due to decreased insulin secretion from *Vhlh-*deficient ß-cells.

**Figure 5:**
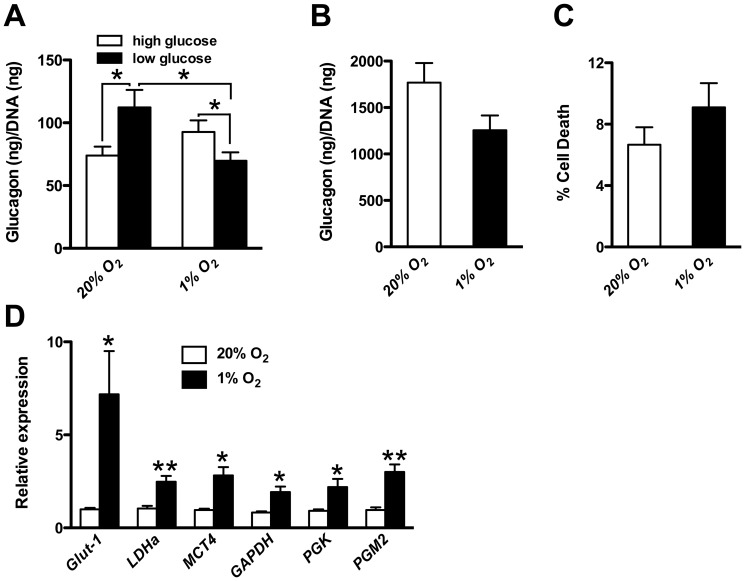
Hypoxia impairs glucagon secretion from α-TC6.1 cells. **A.** Glucagon secretion from α-TC6.1 cells cultured under hypoxic conditions (n = 22) is reduced upon incubation in low glucose as compared to cells cultured under normoxic conditions (n = 21 technical replicates of 3 independent experiments). *p<0.05. **B.** Total glucagon content is not reduced in the cells under hypoxia (n = 6 per condition). **C.** There is no significant change in cell viability, as assessed by propidium iodine uptake, between normoxic and hypoxic conditions (n = 9 per condition). **D.** Gene expression analysis of hypoxia targets revealed an upregulation under hypoxic conditions (n = 6 per condition). Error bars represent standard error of the mean in all cases. *p<0.05, **p<0.005.

Since *Vhlh* is deleted in all endocrine cell types in *Pdx-1-Cre^early^;Vhlh^LoxP/LoxP^* animals, we asked whether the observed α-cell defect was cell-autonomous. Loss of *Vhlh* mimics a hypoxic state, thus to directly ask whether hormone secretion is blocked under conditions of hypoxia, glucagon-producing α-TC6.1 cells were cultured under normal (20%) and reduced (1%) oxygen levels and glucagon secretion assessed. Glucagon secretion in response to low glucose conditions was impaired when cells were incubated in a hypoxic environment (Figure 5A). Total glucagon content was not significantly altered in the cells incubated under hypoxia (Figure 5B). Cell viability was also determined and no significant change in cell death was observed (Figure 5C). As expected, HIF target genes were significantly upregulated upon hypoxia indicating a robust response of the cells to the low oxygen concentration (Figure 5D). Summarily, our results indicate that secretion of glucagon is impaired in islets lacking *Vhlh* and suggest a likely mechanism for the hypoglycemia in *Pdx-1-Cre^early^;Vhlh^LoxP/LoxP^*, *Ngn3-Cre;Vhlh^LoxP/LoxP^* and *Rfx6;Vhlh^LoxP/LoxP^* mice.

## Discussion

Oxygen and HIF activity has been recently implicated in pancreas development [18], [19] and previous work has shown that VHL elimination in pancreatic progenitor cells causes early postnatal lethality [20]. Using the same pancreatic *Pdx-1-Cre^early^* line [Tg(Pdx1/cre)89.1Dam] [8] described in this study to inactivate *Vhlh*, we also observed significant postnatal death in *Vhlh*-deficient mice. In contrast, *Vhlh* inactivation in pancreatic progenitor cells using a different *Pdx-1-Cre* strain [Tg(Pdx1-cre)1Herr] [21] does not result in postnatal lethality [3]. This apparent discrepancy might be due to differences in timing and/or level of Cre expression between the mouse strains. The *Pdx-1-Cre^early^* line used both by us and Shen et al [7] is active earlier during embryogenesis, while Cre expression occurs at a later stage of development in the Tg(Pdx1-cre)1Herr line [9], making it likely that the excision efficiency and cell types affected are distinct in the two models.

It has been suggested that reduced postnatal survival in *Pdx-1-Cre^early^;Vhlh^LoxP/LoxP^* mice might be associated with exocrine defects [7]. Although older surviving *Pdx-1-Cre^early^;Vhlh^LoxP/LoxP^* mice display exocrine lesions, histological examination of pancreata in young mice do not reveal any apparent exocrine defects ([7] and our own results). Our results point to defects in endocrine function as the cause of death of *Pdx-1-Cre^early^;Vhlh^LoxP/LoxP^* pups. Using exocrine- and endocrine-specific Cre-expressing mouse strains, our studies clearly demonstrate that VHL elimination in the endocrine, but not in the exocrine, lineage results in postnatal lethality.

Severe hypoglycemia appears to be the primary cause of death in *Pdx-1-Cre^early^;Vhlh^LoxP/LoxP^* (and *Rfx6-Cre;Vhlh^LoxP/LoxP^* and *Ngn3-Cre;Vhlh^LoxP/LoxP^*) pups. These mice show a marked reduction in glucose levels since the first day of life. Abnormal feeding does not appear to be the cause for the low blood glucose levels, since milk was observed in the stomach of pups and they appeared to be properly nursed by the mother. In agreement with this, surviving mice do not show low blood glucose levels (data not shown). The hypoglycemic phenotype was somewhat surprising given our previous observation that ß-cell-specific knockout of *Vhlh* does not result in fatal hypoglycemia. Inactivation of *Vhlh* in islets results in defects in glucose-stimulated insulin secretion, similar to what has been described in ß-cell-specific *Vhlh* deficient mice, but an increase in insulin that might explain the hypoglycemic phenotype was not detected. These observations suggest that hypoglycemia in young mice with an islet-specific *Vhlh* deficiency is not primarily linked to defects in ß-cell function. We propose that aberrant glucagon secretion may underlie hypoglycemia in mice with VHL loss in neonatal islets. In agreement with this hypothesis, several studies have linked impaired α-cell formation or glucagon production to severe hypoglycemia and associated neonatal lethality [15], [16], [17]. However, it should be noted that other studies have not observed hypoglycemia or postnatal lethality in mice with disrupted glucagon production [22], [23], [24]. The reasons for these discrepancies are not clear but may be related to the different mouse models used.

Several lines of evidence from our study indicate that activation of the hypoxia/HIF pathway in glucagon-producing cells blocks glucagon secretion. First, no increase in serum glucagon levels was detected under fed or fasted conditions in islet-specific *Vhlh* mutant mice despite decreased blood glucose levels. Second, studies performed in isolated islets from islet-specific neonatal *Vhlh* mutant mice revealed defective glucagon secretion. Finally, glucagon-producing α-TC6.1 cells grown under hypoxic conditions failed to secrete glucagon in response to low glucose. Of note, under hypoxic conditions, canonical target genes of the HIF complex are upregulated in α-TC6.1 cells, similar to what we observe in VHL-depleted islets. Our results are in agreement with a recent study performed in isolated islets cultured under hypoxia [25]. This study reported that islets under hypoxia exhibit inappropriate high basal glucagon release. Interestingly, we observed a trend towards increased basal glucagon secretion in *Pdx-1-Cre^early^;Vhlh^LoxP/LoxP^* mutant islets. However, the difference in glucagon secretion between control and mutant islets was not statistically significant despite the relatively large sample size. Significantly, a failure to secrete glucagon was clear when the mutant islets were shifted to low glucose. In apparent contradiction to our hypothesis identifying glucagon-producing cells as the culprit for hypoglycemia and postnatal lethality of islet-specific *Vhlh* mutant mice, transgenic mice with VHL inactivation specifically in α-cells survive to adulthood [7]. Our attempts at reproducing this study were unsuccessful, as the level of gene excision achieved with another *Glucagon-Cre* mouse line [21] was exceedingly low in our hands (data not shown). Nonetheless, it is important to note that the *Glucagon-Cre* line used by Shen et al efficiently targets α-cells only at adult stages, not during early postnatal stages [26]. Thus, a possible explanation for this apparent discrepancy is that VHL inactivation affects glucagon secretion only during early stages. To this regard, it is interesting to note that neonatal pancreatic endocrine cells appear to be functionally immature [27]. It is tempting to speculate that this immaturity can make endocrine cells more sensitive to increased HIF activity. In agreement with this notion, we observed that *Vhlh* inactivation in pancreatic progenitor cells resulted in delayed ß-cell differentiation. Nevertheless, we cannot formally rule out that a combined perturbation in α- and ß-cell function may cause the hypoglycemia observed in *Pdx-1-Cre^early^;Vhlh^LoxP/LoxP^* mice. In the absence of glucagon counter-regulation, mutant mice would effectively have sufficient insulin to reduce glucose levels, thus providing a possible explanation for the hypoglycemia. Indeed, hypoglycemia has been observed in certain models of α- and ß-cell dysfunction. Mice deficient in a component of the voltage-gated sodium channel (VGSC) present with hypoglycemia associated with defective insulin and glucagon secretion [28] and intra-islet insulin signaling is known to play an essential role in the regulation of glucagon secretion in both normo- and hypoglycemic conditions [29]. Interestingly, the inhibitory effect of insulin on glucagon secretion is not observed in *Vhlh*-deficient islets indicating that VHL loss might compromise intra-islet insulin signaling.

A potential concern of our study is the extra-pancreatic Cre expression of *Pdx-1-Cre^early^* and *Ngn3-Cre* mouse strains, including the brain and duodenum. Although we cannot exclude this possibility since no truly pancreas-specific Cre line is available, we consider the likelihood of a non-pancreatic effect causing the lethality as small. First, cell-autonomous defects are detected in islets of *Pdx-1-Cre^early^;Vhlh^LoxP/LoxP^* mice in *in vitro* secretion assays (Figures 3 and 4). Second, no brain expression is observed in the *Rfx6-Cre* mouse strain [12]. Third, a detailed analysis of the brain cell types led to the conclusion that distinct, non-overlapping neurons are marked by *Pdx-1-Cre^early^* and *Ngn3-Cre* lines [11]. It is unknown whether there is overlap in the duodenal expression of *Pdx-1-Cre^early^* and *Ngn3-Cre* line. However, specific loss of VHL in the intestine does not result in any major defects in mice [30].

In contrast to insulin secretion, the mechanism of glucagon secretion is still poorly understood [31]. Our results reveal hypoxia/HIF signaling as a novel regulator of glucagon secretion and further underscore the importance of finely balanced hormone signaling for proper glucose homeostasis.

## Materials and Methods

### Mice

The animal experiments described in this study were approved by the Committee on Animal Research at the University of California, San Francisco (approval number AN088473-01C). Mice were examined after birth daily for signs of ill health. Any animal displaying body condition score 2 (underconditioned, segmentation of vertebral column evident and dorsal pelvic bones palpable) or less was euthanized. Euthanasia in animals <10 days old were performed by cervical dislocation by trained personnel. In animals >10 days old euthanasia was performed by carbon dioxide inhalation followed by cervical dislocation according to UCSF Institutional Animal Care and Use Committee (IACUC) guidelines. *Ins-Cre* and *Ptf1a-Cre* mice were obtained from Dr. Herrera and Dr. Wright respectively [13], [21]. *Pdx-1-Cre^early^, Ngn3-Cre, Rfx6-Cre* and *Vhlh^loxP/loxP^* mice have been described previously [8], [32], [33]. Control animals include Cre-positive animals that are heterozygous for the *Vhlh* floxed allele, and Cre-negative animals that are either heterozygous or homozygous for the *Vhlh* floxed allele. Both genders were included is all analyses.

### Histology, immunofluorescence and gene expression analysis

Pancreata were processed for hematoxylin/eosin and immunostaining as described previously [2]. The following primary antibodies were used: rabbit anti-amylase, 1:700; mouse anti-glucagon 1∶8000 (Sigma Chemical Co, St. Louis, MO); guinea pig anti-insulin, 1∶300, rabbit anti-glucagon, 1∶300 (Linco Research Inc.); armenian hamster anti-Mucin-1, 1∶200 (Neomarkers, Fremont, CA); rabbit anti-Hif1α 1∶500 (Novus Biologicals, Littleton, CO); rabbit anti-somatostatin, 1∶200 (Dako, Carpinteria, CA). Primary antibodies were detected with Alexa-488, Alexa-546, and Alexa-633 conjugated secondary antibodies (Jackson Immunoresearch Laboratory, West Grove, PA). For anti-HIF1α detection, a tyramide Signal Amplification kit (PerkinElmer, Waltham, MA) was used following manufacturer’s instructions. Bright field images were acquired using a Zeiss Axio Imager D1 microscope. A Zeiss Axioscope2 widefield microscope was used to visualize and photograph fluorescence. Confocal images were acquired using an LTC SP2 confocal microscope (Leica).

RNA isolation, cDNA preparation, and quantitative PCR were performed as described [34]. Gene expression was normalized to cyclophilin and actin expression. Primer sequences are available upon request.

### Glucose tolerance tests and hormone measurement

Analyses on hypoglycemic mice were carried out between p13 and p18 after birth. After a 16hr fast for adults or 6-hour fast for pups (<21days), animals were weighed, blood glucose measured followed by an intraperitoneal injection of glucose as described previously [2]. For *in vivo* hormone measurement, blood from the tail vein was centrifuged to collect serum, and insulin and glucagon concentration was calculated using the Insulin EIA kit (ALPCO) and Glucagon RIA kit (#GL-32K, Millipore) respectively. An overnight fast was performed in pups aged p15 for assessing fasted glucagon levels in serum.

### Islet isolation and *in vitro* insulin and glucagon secretion

The Islet Production Facility Core at the Diabetes Center at UCSF isolated mouse islets. Glucose stimulated insulin secretion from isolated islets and measurement of insulin content of islets have been described previously [2], [35]. For glucagon secretion, islets (10 per replicate) were incubated in 500 µl of high (25 mM) or low (2.7 mM) glucose at 37°C for one hour. Supernatants were collected, proteinase inhibitors added (Aprotinin, Sigma Chemical Co, St. Louis, MO) and assayed for glucagon using a Glucagon Elisa Kit (Yanaihara Institute Inc, Shizouka, Japan). Total protein content was measured using a DC Protein Assay kit (Bio-Rad, Hercules, CA). To analyze the inhibitory effects of insulin on glucagon secretion from isolated islets, exogenous insulin was added at a final concentration of 17 nM. For the hypoxia experiments, α-TC6.1 murine cells (obtained from ATCC, catalog number CRL-2934) were grown in low glucose medium (2 g/l glucose) in a hypoxia incubator (1% O_2_ 37°C and 5% CO_2_) for 12 hours. For the glucagon secretion assay, cells were incubated in KRB-high glucose (25 mM) at 37°C or KRB-low glucose (2.7 mM) for one hour. The hypoxia/normoxia condition was maintained during this incubation period. Supernatants were collected, proteinase inhibitors added and assayed for glucagon as described above. Cellular DNA was extracted with ATE (Ammonium Hydroxide/Triton X-100) solution and quantified using Nanodrop-2000 (Thermo Scientific).

### Glucagon stimulation test

Glucagon (Sigma) was injected intraperitoneally at 16 µg/kg body weight and blood glucose monitored for 2 hours using a Lifescan Glucometer.

### Statistical analysis

Student’s t test was used for calculating significance for all data sets except for the glucagon secretion experiments. The values in these experiments were not normally distributed and were compared using the Mann-Whitney U test, calculated according to the Monte Carlo method. Differences were considered significant if p<0.05.

## Supporting Information

Figure S1 **A.** Survival curve of *Pdx-1-Cre^early^;Vhlh^LoxP/LoxP^* mice (n = 35) as compared to control littermates (n = 40). **B.** Normal islet formation in *Rfx6-Cre;Vhlh^LoxP/LoxP^* mice.
(TIF)

Figure S2 **A.** *Ptf1a-Cre* is not efficiently expressed in the pancreatic islet. The Z/AP reporter strain [36], which expresses alkaline phosphatase (AP) upon Cre-mediated recombination, was used to determine the Cre recombinase activity in *Ptf1a-Cre* mice. Staining for alkaline phosphatase marks cells that have undergone recombination in 4 week old *Ptf1a-Cre;Z/AP* mice. Histological sections were enzymatically stained for alkaline phosphatase activity (blue), DBA lectin to mark pancreatic ducts (brown) and nuclear fast red as a counterstain (pink). Exocrine tissue is efficiently targeted in *Ptf1a-Cre;Z/AP* pancreas as shown by the clear alkaline phosphatase. However, only scattered cells are marked by alkaline phosphatase in islets (dashed in yellow). **B.** Robust HIF1α accumulation in exocrine, but not endocrine, pancreatic tissue of *Ptf1a-Cre;Vhlh^LoxP/LoxP^* mice (p15). Islets are outlined in yellow. **C.** Normal blood glucose levels in neonatal (left) and older (right) *Ptf1a-Cre;Vhlh^LoxP/LoxP^* mice.
(TIF)

Figure S3 **Glucagon stimulation in** ***Pdx-1-Cre^early^;Vhlh^LoxP/LoxP^*** **mutant mice.** Exogenous glucagon was administered to control (square, n = 17) and *Pdx-1-Cre^early^;Vhlh^LoxP/LoxP^* (triangle, n = 7) animals and blood glucose measured over the next two hours. **p<0.01, ****p<10^−6^.
(TIF)
