# Supplementary figures and images for: Elimination of Von Hippel-Lindau Function Perturbs Pancreas Endocrine Homeostasis in Mice
Source: PLoS One. 2013 Aug 20;8(8):e72213. doi: 10.1371/journal.pone.0072213 (PMC3748057; doi:10.1371/journal.pone.0072213)

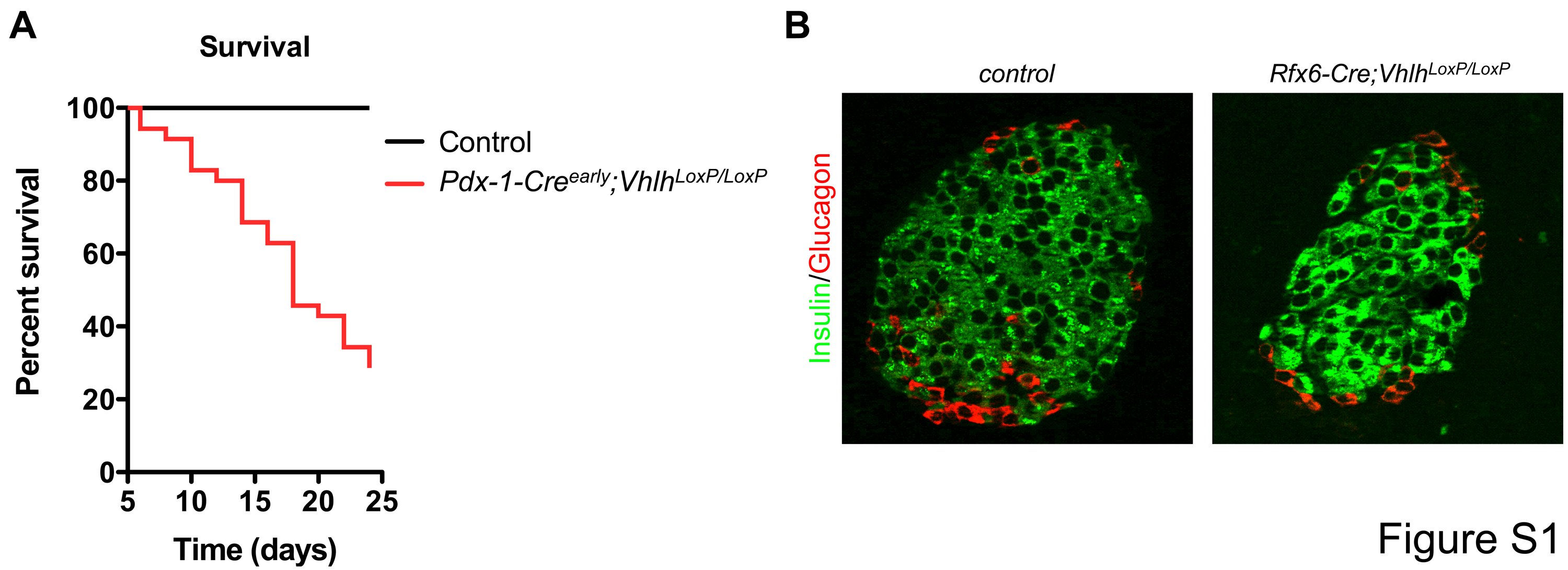

Supplement: Figure S1 — A. Survival curve of Pdx-1-Creearly;VhlhLoxP/LoxP mice (n = 35) as compared to control littermates (n = 40). B. Normal islet formation in Rfx6-Cre;VhlhLoxP/LoxP mice. (TIF) [file pone.0072213.s001.tif]

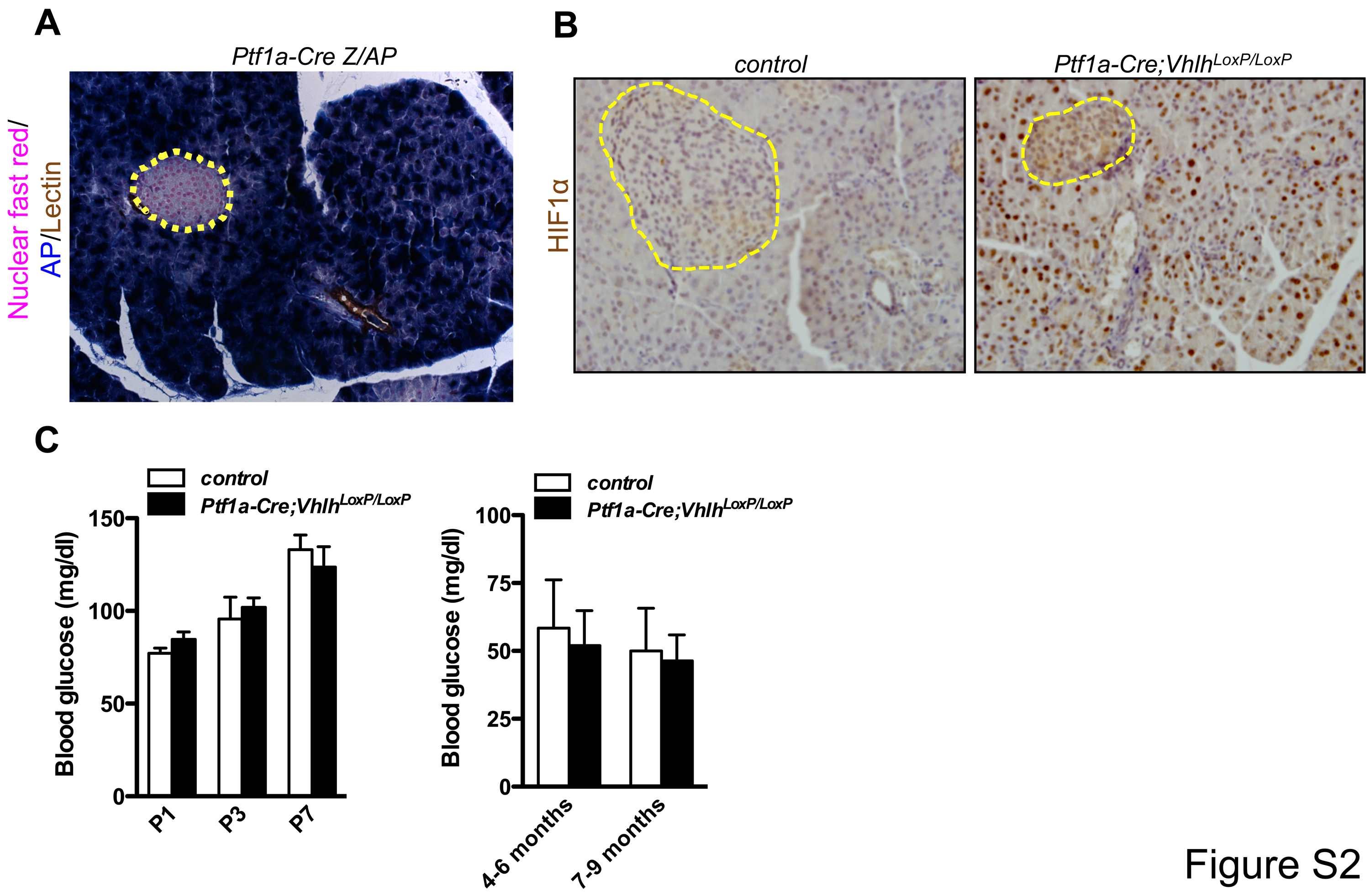

Supplement: Figure S2 — A. Ptf1a-Cre is not efficiently expressed in the pancreatic islet. The Z/AP reporter strain [36], which expresses alkaline phosphatase (AP) upon Cre-mediated recombination, was used to determine the Cre recombinase activity in Ptf1a-Cre mice. Staining for alkaline phosphatase marks cells that have undergone recombination in 4 week old Ptf1a-Cre;Z/AP mice. Histological sections were enzymatically stained for alkaline phosphatase activity (blue), DBA lectin to mark pancreatic ducts (brown) and nuclear fast red as a counterstain (pink). Exocrine tissue is efficiently targeted in Ptf1a-Cre;Z/AP pancreas as shown by the clear alkaline phosphatase. However, only scattered cells are marked by alkaline phosphatase in islets (dashed in yellow). B. Robust HIF1α accumulation in exocrine, but not endocrine, pancreatic tissue of Ptf1a-Cre;VhlhLoxP/LoxP mice (p15). Islets are outlined in yellow. C. Normal blood glucose levels in neonatal (left) and older (right) Ptf1a-Cre;VhlhLoxP/LoxP mice. (TIF) [file pone.0072213.s002.tif]

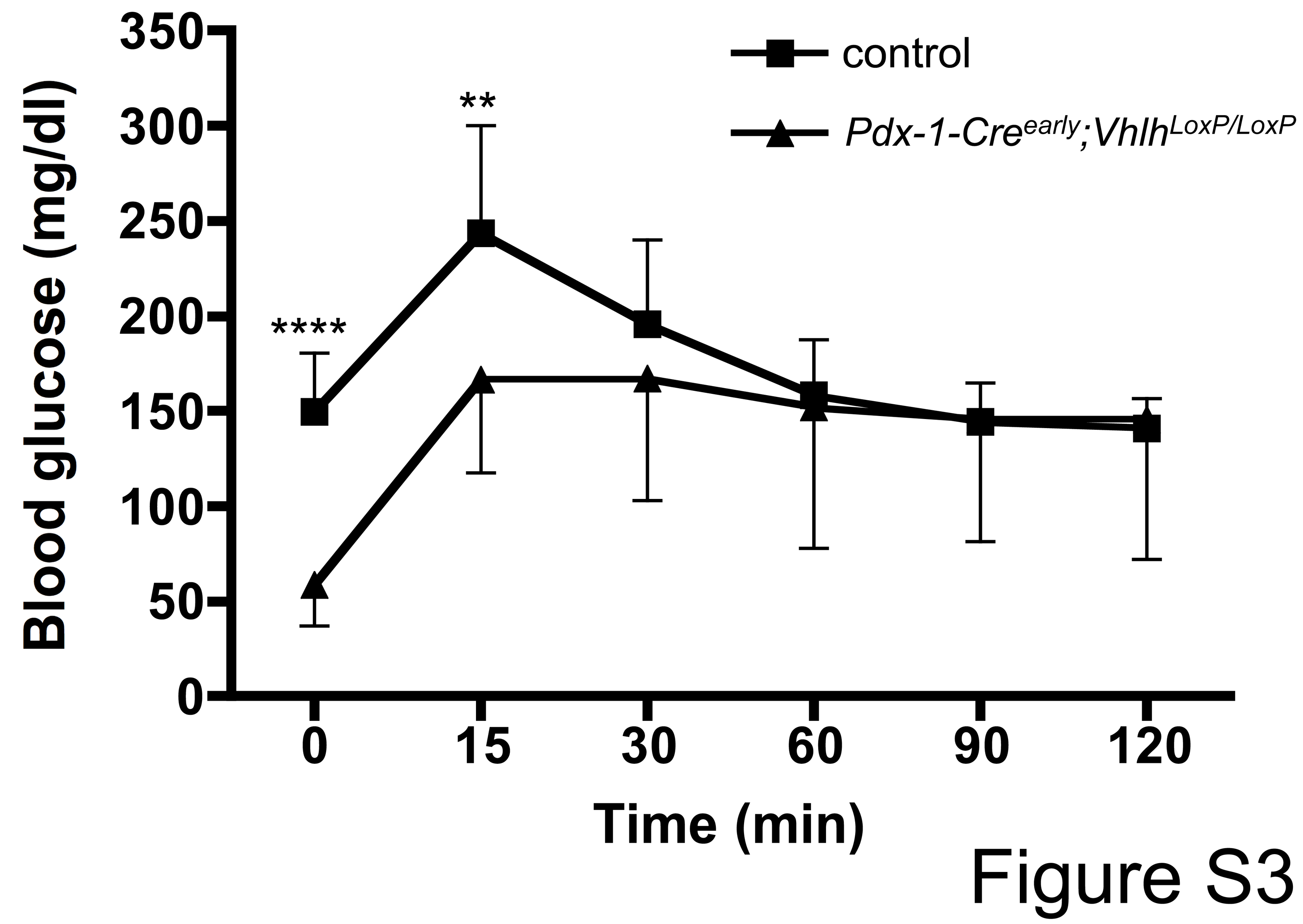

Supplement: Figure S3 — Glucagon stimulation in Pdx-1-Creearly;VhlhLoxP/LoxP mutant mice. Exogenous glucagon was administered to control (square, n = 17) and Pdx-1-Creearly;VhlhLoxP/LoxP (triangle, n = 7) animals and blood glucose measured over the next two hours. **p<0.01, ****p<10−6. (TIF) [file pone.0072213.s003.tif]
